# Studies on the Mechanism of the Volatile Oils from Caoguo-4 Decoction in Regulating Spleen Deficiency Diarrhea by Adjusting Intestinal Microbiota

**DOI:** 10.1155/2022/5559151

**Published:** 2022-01-27

**Authors:** Li Mei, Fang Wang, Ming Yang, Zhiyong Liu, Liangfeng Wang, Qingyao Chen, Fengqin Li, Xiaofei Zhang

**Affiliations:** ^1^Jiangxi University of Chinese Medicine, 1688 Meiling Avenue, Wanli District, Nanchang 330004, China; ^2^Inner Mongolia Minzu University, No. 536 West Huolinhe Street, Horqin District, Tongliao Inner Mongolia Autonomous Region 028000, China; ^3^College of Pharmacy, Shanghai University of Chinese Medicine, Shanghai 201203, China; ^4^Shaanxi University of Chinese Medicine, Xixian Avenue, Xixian District, Xianyang 712046, China

## Abstract

**Background:**

The Caoguo-4 decoction, a classical Mongolian medicine formula, is widely used to treat spleen deficiency diarrhea (SDD) in Mongolian for decades. Previously, the Caoguo-4 decoction volatile oil has been confirmed to be effective in ameliorating symptoms of spleen deficiency diarrhea in an animal model. However, the underlying mechanism of the Caoguo-4 decoction volatile oil is yet to be established. The aim of the current study was to investigate the antidiarrheal effects and mechanism of the Caoguo-4 decoction volatile oil.

**Method:**

Wistar rats were randomly divided into 5 groups of 10 animals including control, model, positive, Caoguo-4 decoction, and Caoguo-4 decoction volatile oil groups (10 rats in each group). All the rats, besides those in the control group, were induced to develop SDD by a bitter-cold purgation method with Xiaochengqi decoction. The antidiarrheal effect of Caoguo-4 decoction volatile oil was evaluated by pathological section, serum D-xylose and AMS content, plasma MTL content, and gut microbiota analysis via 16S rRNA sequencing.

**Results:**

The results showed that the developed SDD rat model (model group) had decreased food intake, increased weight loss, soft stool, and bad hair color. When compared with the control group, serum was significantly reduced serum D-xylose and AML but increased MTL levels in the model group (*p* < 0.05). However, after treatment with either the Caoguo-4 decoction (the decoction group) or Smecta (the positive group) or volatile oil from the Caoguo-4 decoction (the volatile oil group), a significant increase in the serum D-xylose levels was observed. Additionally, AML levels significantly increased in the positive and volatile oil groups, and MTL levels significantly decreased in the decoction and volatile oil groups, when compared with the model group (*p* < 0.05). The pathological changes of the intestinal mucosa showed that the structure of the epithelium in the villi of the small intestine was affected, deformed, and incomplete in the model group when compared with the control group. However, either the decoction group or the volatile oil group recovered the villous morphology. The results of OTU analysis and alpha diversity analysis of intestinal bacteria showed that the intestinal microbiota of the SDD model rats showed an obvious decrease in richness and diversity of intestinal microbiota. But the intervention treatment of decoction and volatile oil could significantly recover the richness and diversity of intestinal microbiota.

**Conclusion:**

The intestinal microbiota destroyed in SDD modelling could be significantly improved by the Caoguo-4 decoction volatile oils, which provides reference for clinical medication.

## 1. Introduction

Spleen deficiency diarrhea (SDD) is a common disease of traditional Chinese medicine (TCM). The main cause of SDD according to TCM is considered to be spleen deficiency, endophytic dampness, Qi consumption, and stomach disharmony, resulting in conductive dysfunction of the intestines [1, 2]. The deficient spleen fails to transport and the weak stomach fails to digest and process foods; thus, the water and foods are then retained to affect the ascent of spleen yang; consequently, it collapses to cause diarrhea [3]. At present, the specific pathogenesis of SDD remains unknown: immune dysfunction, intestinal barrier dysfunction, mental factors, etc. [4]. With the deepening of research, gut microbiota has become one of the most critical factors in SDD progression, accompanied by diarrhea induced by dysfunctional or infection intestinal environment [5, 6]. Related research also confirmed that the quantity of beneficial physiological bacteria such as *Lactobacillus* and *Bifidobacterium* in the intestine of mice is negatively correlated with the severity of the symptoms of SDD in mice; that is, the more severe the symptom of SDD in mice, the smaller the quantity of *Lactobacillus* and *Bifidobacterium* in the intestine of mice, leading to disorder of gut microbiota [7]. Therefore, a better understanding of the gastrointestinal microflora in SDD has been of great significance. Acupoint application therapy is the application of Chinese herbal medicine to the corresponding points to regulate meridians, yin and yang, and qi as well as the blood of the human body [8]. The Shenque point has a thin cuticle making it conducive to rapid drug absorption, and its location close to the intestinal tract strengthens the spleen and stomach by promoting the flow of Qi and relaxing the bowels [9]. Increasing evidence suggests that TCM achieved a good therapeutic effect for SDD for its less toxicity and equal or even better antidiarrheal efficacy [10]. Caoguo-4 decoction is a kind of traditional Mongolian medicine formula, originated from the medical classic of “Yi Fang Feng Ji.” It has long been used clinically for the maintenance of “Heyi” and upward “Heyi” disease and specifically has been identified to have a significant effect on spleen deficiency diarrhea. “Mongolian Medicine Prescription” [11]: “Used for the treatment of stomach “Heyi “syndrome, head tingling, spleen disease, etc.” “Chinese Medical Encyclopedia (Mongolian version)” recorded [12]: “Indications for head stabbing pain, abdominal distension and bowel rumbling caused by Ascending “Heyi” and “Siming Heyi.” “It is composed of *Amomum tsao-ko* Crevost et Lemarie, *Eugenia caryophyllata* Thunb., *Aucklandia lappa* Decne., and *Foeniculum vulgare Mill.*, which all belong to the “warm-natured” category of the Mongolian medicine, with a role specifically in dispelling the spleen and stomach chill. However, Caoguo-4 decoction has a curative clinical effect on SDD, but with limited development due to being short of defined pharmacodynamics and mechanisms.

Volatile oils refer to the active components from TCM. Valuable properties of volatile oils have been confirmed experimentally or empirically. Examples include [13] *Eugenia caryophyllata* Thunb. (strengthening the spleen and resisting bacteria), *Aucklandia lappa* Decne. (moving Qi and relieving pain), *Amomum tsao-ko* Crevost et Lemarie (invigorating the stomach and dispelling dampness), *Foeniculum vulgare* Mill. (regulating Qi flowing for strengthening the spleen), etc. In the previous study from this group, the Caoguo-4 decoction volatile oil has been confirmed to be effective in ameliorating symptoms of the SDD rat model, which is comparable to the effect of the Caoguo-4 decoction. However, the underlying mechanisms by which the Caoguo-4 decoction volatile oil exhibits antidiarrheal activity remains poorly understood. Hence, the aim of this study was to investigate the antidiarrheal effect and mechanism of the Caoguo-4 decoction volatile oil through regulation of intestinal microbiota.

## 2. Materials and Methods

### 2.1. Materials and Chemicals

Amomum tsao-ko (batch number: 1709009), cloves (batch number: 1710004), fennel (batch number: 1711009), Costustoot (batch number: 1703003), Rheum officinale (batch number: 1806007), fructus aurantii immaturus (batch number: 1806008), and *Magnolia officinalis* (batch number: 1805003) were purchased from Jiangxi Zhangshu Tianqitang Chinese Herbal Slices Co., Ltd. (Jiangxi, China). The commercial enzyme-linked immunosorbent assay (ELISA) kits for D-xylose, serum motilin (MTL), and amylase were purchased from Jiancheng Enzymatic Immunity Co., Ltd. (Nanjing, China). Jojoba oil was purchased from Dimei Biotechnology Co., Ltd. (Guangzhou, China).

### 2.2. Plant Materials and Volatile Oil Extraction

Caoguo-4 decoction, a kind of traditional Mongolian medicine formula, contains the following ingredient: *Amomum tsao-ko* Crevost et Lemarie (also known as CaoGuo in China), *Eugenia caryophyllata* Thunb. (also known as DingXiang in China), *Aucklandia lappa* Decne. (also known as MuXiang in China), and *Foeniculum vulgare* Mill. (also known as HuiXiang in China). All the herbs were purchased from Chengdu Huichu Technology Co., Ltd. (Chengdu, China), which were authenticated by Professor Fei Ge (Jiangxi University of Traditional Chinese Medicine, Nanchang, China). According to the ratio of CG : MX : DX : HX = 5 : 5 : 3 : 3, the dried Chinese herbs were crushed into coarse powder.

Caoguo-4 decoction was prepared as follows: 100 g crude Caoguo-4 decoction was made into powder and put in 10 times of water for 15 minutes, boiled for 30 minutes, filtered, and collected. Repeat such procedures. Puted the two liquid together, concentrated them to 0.256 g/ml.

Caoguo-4 decoction volatile oil was extracted by the steam distillation method. The steam distillations were carried out according to the Chinese Pharmacopoeia (2020). Briefly, 360 g of Caoguo-4 decoction medicinal powder was completely immersed in Milli-Q water in proportion of 1 : 10 (*W*/*V*) for 30 min. The mixture was submitted to hydrodistillation in a Clevenger-type apparatus at temperature of 100°C for 240 minutes. Volatile oil was dried over Na_2_SO_4_. The yield of volatile oil was 5% (*V*/*W*), and the oil was stored at -20°C until analysis. For animal experiment, the volatile oil was diluted with jojoba oil with the dilution ratio of 1 : 10 (*V*/*V*).

### 2.3. Animal Experimental Design

#### 2.3.1. Animal

SPF Wistar rats weighing ∼200–220 g were purchased from Lai-Ke-Jing-Da Experimental Animal Co. Ltd. (Hunan, China) and consisted of both genders at a proportion of 1 : 1. The animals were housed individually in the Experimental Animal Center of Jiangxi University of Chinese Medicine (Jiangxi, China). Standard tap water and rat food were available. After acclimation for 7 days, all rats were randomly divided into 5 groups of 10 animals each as follows: the normal control group (CG), the spleen deficiency diarrhea model group of treatment groups (MG), the positive group of treatment groups (YXG), the Caoguo-4 decoction group of treatment groups (TG), and the Caoguo-4 decoction volatile oil group of treatment groups (HG). The spleen deficiency diarrhea model was induced in the MG group, the YXG group, the TG group, and the HG group.

During all experiments, water and food were available ad libitum. All animal procedures were conducted in accordance with the institutional guidelines approved by the Ethics Committee of the Jiangxi University of Chinese Medicine (no. JZLLSC2018-0086).

#### 2.3.2. Spleen Deficiency Diarrhea Model and Group Treatments

The spleen deficiency diarrhea (SDD) model was established using a bitter-cold purgation method with Xiaochengqi decoction [14, 15]. Briefly, the rats were intragastrically administered with 3 ml/100 g Xiaochengqi decoction for 21 days once every other day. Meanwhile, the rats were not fed with any chow but drinking water freely on gavage day and were fed ad libitum on the next day. The CG group was intragastrically administered with the same volume of double-distilled water with food and water ad libitum. The YXG group was intragastrically administered with Smecta, prepared in 40 mg/ml suspension with distilled water (equivalent to 10 times the clinical dosage). The TG group was given the Caoguo-4 decoction with a mass concentration of 0.256 g/ml (the amount of 10 ml/kg/day was applied). The HG group was given the Caoguo-4 decoction volatile oil through the umbilical cord and stimulating the Shenque acupoint with 1.92 ml (the yield of volatile oil was 5% (*V*/*W*), 10 times of the oral dose). The modelling was considered successful if the following criteria were meet: (a) slow weight gain or weight loss, (b) less eating amount and more drinking volume, (c) loose stools, (d) lassitude or irritating, (e) yellow and dry hair, (f) curling up for fear of cold, and (g) D-xylose and amylase (AMS) levels measured once per week for 3 weeks.

Xiaochengqi decoction, a kind of traditional Chinese formula, contains the following ingredient: *Radix et Rhizoma Rhei*, *Fructus Citri aurantii immaturus*, and *Cortex Magnoliae officinalis* with the amount ratio of 2 : 3 : 3 for SDD modelling. All the herbs were purchased from Jiangzhong Chinese Medicine Yinpian Co. Ltd. (Jiangxi, China) as crude herbs. Xiaochengqi decoction was prepared as follows: the herbs were soaked in 10 times the amount of water for 30 minutes and heated for 1 hour each time, and the filtrate was combined twice and then condensed to 1 g/ml.

### 2.4. Sample Collection

At the 21^th^ day, after fasting for 24 hours, the rats were sacrificed by intraperitoneal injection of 1% pentobarbital sodium (50 mg pentobarbital/kg rat body weight). Blood was harvested from the abdominal aorta into tubes with ethylene diamine tetra acetic acid. Plasma was isolated by centrifugation at 4000 rpm for 15 min at 4.0°C. The supernatant was collected and stored at -80°C for later analysis. The small intestine was put into 4% buffered formalin solution and embedded in paraffin. These sections were stained with hematoxylin and eosin (HE) and periodic acid-Schiff (PAS), respectively, in accordance with the standard procedures for histopathological analysis. The intestinal feces were taken under sterile conditions and then put into sterile tubes in liquid nitrogen for further analysis. The amounts of spleen deficiency-associated cytokines including D-xylose, AMS, and MTL were determined using ELISA kits according to the manufacturers' instructions.

### 2.5. ELISA

The rats were sacrificed by intraperitoneal injection of 1% pentobarbital sodium (50 mg pentobarbital/kg rat body weight). Serum was stored at −80°C for analysis of D-xylose and AMS content using the enzyme-linked immunosorbent assay (D-xylose ELISA kit provided by Nanjing Jiancheng Bioengineering Institute Co., Ltd., no.20180621; AMS ELISA kit provided by Nanjing Jiancheng Bioengineering Institute Co., Ltd., no. 20180612). Plasma was stored at −80°C for analysis of MTL content using the enzyme-linked immunosorbent assay (MTL ELISA kit provided by Nanjing Jiancheng Bioengineering Institute Co., Ltd., no. 20180901).

### 2.6. Histopathological Observation

The jejunum tissues were isolated, rinsed with ice PBS, fixed in 4% paraformaldehyde solution for 24 hours, removed, cut well into the dewatering box, and dehydrated in 75% alcohol for 4 h, 85% alcohol, and 90% alcohol for 2 hours each; anhydrous ethanol I and anhydrous ethanol II for 30 min; alcohol benzene, xylene I, and xylene II for 5-10 min; and wax I, wax II, and wax III for 1 h. The tissue was soaked in wax and embedded. The paraffin tissues were cut into 4 *μ*m thick sections with a cryotome and were stained with hematoxylin and eosin (H&E), periodic acid-Schiff (PAS), or Masson's trichrome (M-T). The morphological changes in the stained tissues were observed under a light microscope.

### 2.7. Gut Microbiota Analysis

The feces samples collected from all groups on the third week were used for the microbial community analysis. Total genomic DNAs from the feces of mice were extracted using the E.Z.N.A.® stool DNAKit (Omega Bio-Tek, Norcross, GA, U.S.) according to the manufacturer's instruction. The quality of extracted DNA was checked by 1% agarose gel electrophoresis and spectrophotometry (optical density at the 260 nm/280 nm ratio). All extracted DNA samples were stored at -20°C for further analysis.

The V3–V4 hypervariable regions of the 16s rRNA gene were subjected to high-throughput sequencing by Beijing Allwegene Tech, Ltd. (Beijing, China) using the Illumina Miseq PE300 sequencing platform (Illumina, Inc., CA, USA). The V3-V4 regions of the bacterial 16s rRNA gene were amplified with the universal primers of the forward 338F (5′-ACTCCTACGGGAGGCAGCAG-3) 5and the reverse 806R (5′-GACTACHVGGGTWTCTAAT-3′). The PCR program was as follows: 95°C for 5 min and 25 cycles at 95°C for 30 s, 55°C for 30 s, and 72°C for 30 s with the final extension of 72°C for 10 min. PCR reactions were performed in triplicate: 25 *μ*l mixture containing 2.5 *μ*l of 10× Pyrobest Buffer, 2 *μ*l of 2.5 mM dNTPs, 1 *μ*l of each primer (10 *μ*M), 0.4 U of Pyrobest DNA Polymerase (TaKaRa), and 15 ng of template DNA. The amplicon mixture was applied to the MiSeq Genome Sequencer (Illumina, San Diego, CA, USA).

### 2.8. Illumina MiSeq Sequencing

Amplicons were extracted from 2% agarose gels and purified using the AxyPrep DNA Gel Extraction Kit (Axygen Biosciences, Union City, CA, U.S.) according to the manufacturer's instructions and quantified using QuantiFluor™-ST (Promega, U.S.). Purified amplicons were pooled in equimolar and paired-end sequenced (2 × 300) on an Illumina MiSeq platform according to the standard protocols.

The extraction of high-quality sequences was firstly performed with the QIIME package (Quantitative Insights Into Microbial Ecology) (v1.2.1). Raw sequences were selected based on sequence length, quality, primer, and tag. The raw sequences were selected, and the low-quality sequences were removed; specific information is as follows: (i) the 300 bp reads were truncated at any site receiving an average quality score < 20 over a 50 bp sliding window, discarding the truncated reads that were shorter than 50 bp. (ii) Exact barcode matching, 2 nucleotide mismatches in primer matching, and reads containing ambiguous characters were removed. (iii) Only sequences that overlap longer than 10 bp were assembled according to their overlap sequence. Reads which could not be assembled were discarded. The unique sequence set was classified into operational taxonomic units (OTUs) under the threshold of 97% identity using UCLUST. Chimeric sequences were identified and removed using USEARCH (version 8.0.1623). The taxonomy of each 16S rRNA gene sequence was analyzed by UCLUST against the Silva119 16S rRNA database using confidence threshold of 90%.

### 2.9. Statistical Analysis

Statistical analysis was performed using SPSS 24.0. The graphs were made using GraphPad Prism 6.0. One-way analysis of variance (ANOVA) and two-tail Student's *t*-test were conducted for comparisons among the multiple groups. *p* < 0.05 was considered to be statistically significant.

## 3. Results

### 3.1. Macro Characterization

**Figure 1:**
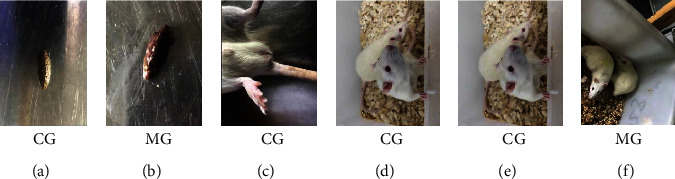
Microcosmic indexes of rats in the control group and the model group. Note: CG: control group; MG: model group: (a) stool from the control group; (b) stool from the model group; (c) anal area of the control group; (d) anal area of the model group; (e) posture of the control group; (f) posture of the model group.

Throughout the experiment, the physiological status between the CG group and the other four groups (MG, TG, HG, and YXG group) is significant. The CG group displayed flexible reaction, smooth coat, controlled food intake, and granular stool. The spleen deficiency model in the other four groups (MG, TG, HG, and YXG group) was developed using Xiaochengqi decoction as previously described. The rats in other groups began to have diarrhea on the 3^rd^ day after the establishment of the model. The water content in their stools increased markedly. On the 7^th^ day, the diarrhea was more obvious. The stools were thin, and there was dirt around the anal area. They squinted visually, crouched obviously, piled up, arched back, were tired and sleepy, fed less, reacted slowly, and defecated many times, and their fur was dark and glossy (Figure 1).

### 3.2. Effect of Caoguo-4 Decoction Volatile Oil on Serum D-Xylose and AMS Content and Plasma MTL Content in Spleen Deficiency Rats

Table 1 shows that the plasma MTL content in the MC group was higher than that in the CG group (*p* < 0.05). The plasma MTL content in the TG and MG groups was lower than that in the MG group (*p* < 0.05). The serum D-xylose and AMS content in the MG group was lower than that in the CG group (*p* < 0.05), but for the TG, MG, and YXG groups, serum was significantly increased in the serum D-xylose and AMS levels (*p* < 0.01 or *p* < 0.05). This result indicated that Caoguo-4 decoction and Caoguo-4 decoction volatile oil could upregulate the content of D-xylose, AML, and MTL in rats.

### 3.3. The Histopathological Changes in the Intestinal Mucosa

**Figure 2:**
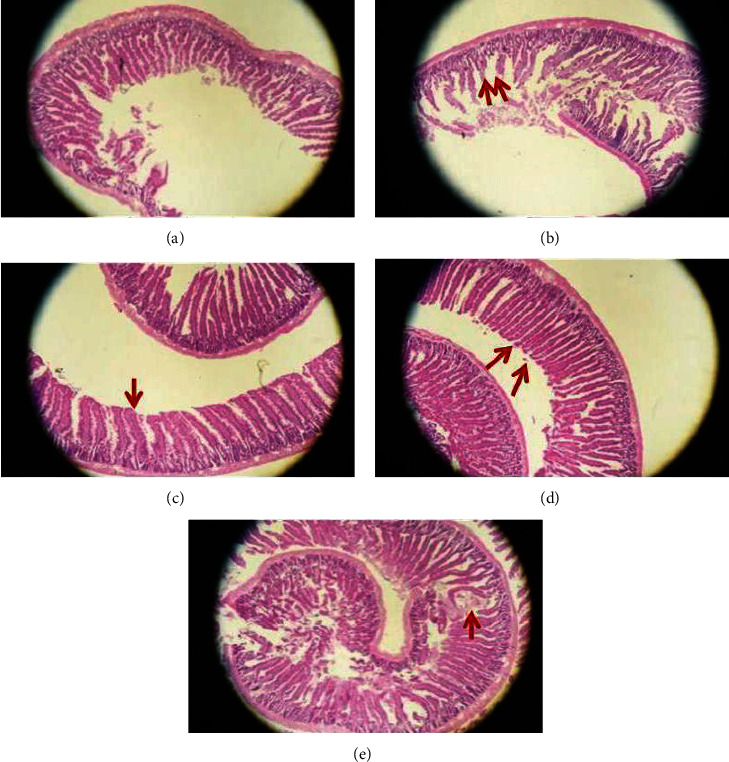
Effect of each group on small intestinal mucosa histopathology in rats with spleen deficiency diarrhea (hematoxylin-eosin; 40x magnification): (a) CG group, (b) MG group, (c) TG group, (d) HG group, and (e) YXG group.

The histopathological changes of the intestinal mucosa of mice in all groups were analyzed by HE staining (Figure 2). Mice in the CG group showed integrity of the intestinal epithelium and no inflammatory cell infiltration in interstitial cells. But in the MC group, mucosal lesions with a massively destructed epithelium and a mass of glands were decreased with disorder and inflammatory cell infiltration. In the TG and HG group, with a little inflammatory cell infiltration, the structure of the epithelium in the villi of the small intestine was almost complete, only partially broken. This result indicated that Caoguo-4 decoction and Caoguo-4 decoction volatile oil could significantly protect intestinal mucosa structure and reduce histologic inflammation.

### 3.4. OTU Analysis

**Figure 3:**
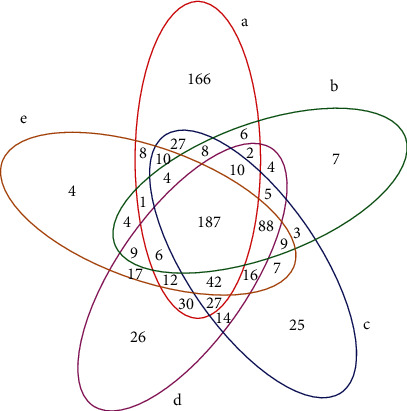
OTU Venn figure: a—CG group, b—MG group, c—TG group, d—HG group, and e—YXG group.

Figure 3 shows the operational taxonomic unit (OTU) number of all the bacteria in the gut. When compared with the CG group, the MG group had 224 identical species, the TG group had 315 identical species, the HG group had 316 identical species, and the TYG group had 270 identical species. Further, when compared with the MG group, the TG group had 314 identical species, the HG group had 311 identical species, and the YXG group had 308 identical species. Between the TG group and the HG group, there were 389 identical species. It can be seen that after the treatment using the Caoguo-4 decoction volatile oil, the number of bacterial OTUs in the HG group is the same as that in the TG group; further, there was similar number of bacterial OTUs in the TG group and the CG group. The results indicate that the simultaneous use of the Caoguo-4 decoction volatile oil and the Caoguo-4 decoction can effectively restore intestinal microbiota and restore intestinal microecological environment.

**Figure 4:**
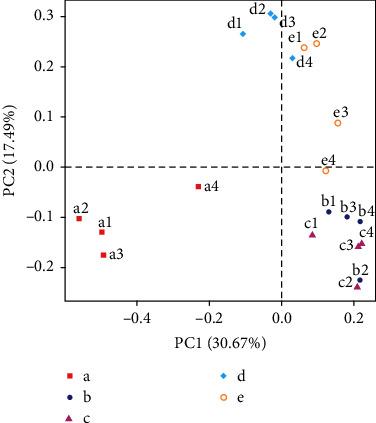
PCA based on OTU level: a—CG group, b—MG group, c—TG group, d—HG group, and e—YXG group.

In Figure 4, we find that the YXG group points are more dispersed, indicating that the intestinal microbial community in this group of rats is the most different. However, the TG group and the HG group points are relatively concentrated, indicating that the difference between the two groups of rat's intestinal microbial community is small. Interestingly, in the HG group, the difference was minimal.

### 3.5. Alpha Diversity Analysis

**Figure 5:**
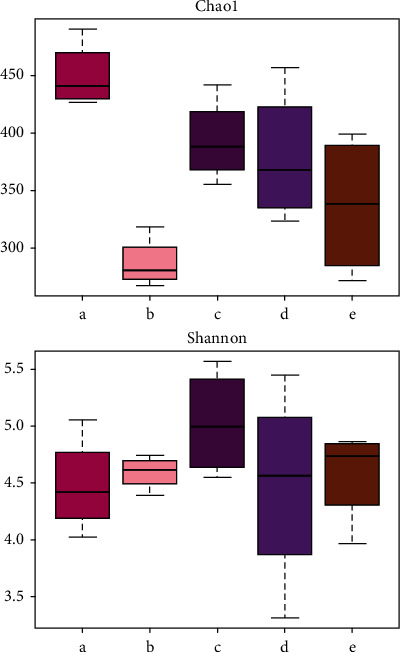
Alpha diversity index box figure: a—CG group, b—MG group, c—TG group, d—HG group, and e—YXG group.

The good coverage value for each group was greater than 0.99, indicating that the results of this sequencing can reflect the actual situation of microorganisms in the sample. The results of alpha diversity analysis between groups (Figure 5) indicated that compared with the CG group, the intestinal microbiota richness index (Figure 5, chao1 and observed species) (Table 2) in the MG group was significantly reduced (*p* < 0.01) and the diversity index (PD whole tree) significantly decreased (*p* < 0.05). Further, compared with the MG group, the intestinal microbiota richness index (Figure 5, chao1 and observed species) of rats in the TG group and HG group was significantly increased (*p* < 0.01) and the diversity index (whole tree) increased significantly (*p* < 0.05).There was no significant difference in the YXG group (*p* > 0.05). Further, there was no significant difference in the Shannon index (Figure 5, Shannon) in each experimental group (*p* > 0.05), which indicated that the intestinal microbiota of spleen deficiency diarrhea rats had been well recovered posttreatment with Caoguo-4 decoction.

### 3.6. Species Annotation Analysis

**Figure 6:**
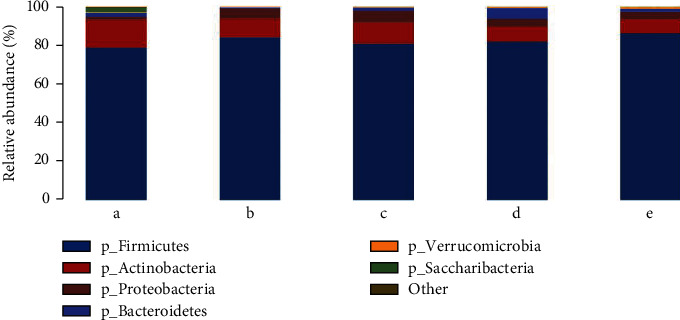
Profiling histograms of species at different groups of phylum classification levels. The horizontal coordinate is the sample name, and the longitudinal coordinates are the relative abundance of the species in the sample: a—CG group, b—MG group, c—TG group, d—HG group, and e—YXG group.

According to the OTUs obtained from the sample sequencing and the species represented by OTUs, the corresponding columnar diagrams of the species were drawn for each sample (Figure 6) at the phylum and belonging to the classification grade.

The distribution of phylum horizontal species is shown in Table 3. The results show that the intestinal structure of rats at the phylum level is mainly thick-walled fungus, *Actinomycetes* phylum, and deformed fungus phylum, in which the proportion of deformed bacteria phylum is the lowest, the proportion of thick-walled fungus is the highest, and *Actinomycetes* phylum is the second most dominant flora. Compared to the control group, in the model group, *Saccharibacteria* (*p* < 0.05), *Tenericutes* (*p* < 0.01), *Euryarchaeota* (*p* < 0.05), and *Cyanobacteria* significantly decreased (*p* < 0.01). However, *Proteobacteria* significantly increased (*p* < 0.01). Compared with the model group, the *Saccharibacteria* of the decoction group increased significantly (*p* < 0.05). And *Proteobacteria* of the volatile oil group decreased significantly (*p* < 0.05), *Saccharibacteria* significantly increased (*p* < 0.05), and there was no significant difference in the positive group.

**Figure 7:**
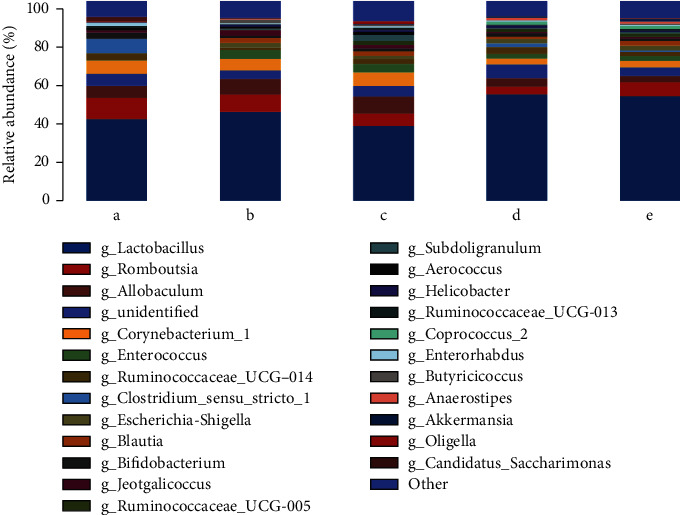
Profiling histograms of species at different groups of genus classification levels: a—CG group, b—MG group, c—TG group, d—HG group, and e—YXG group.

Figure 7 and Table 4 show the distribution of horizontal species; the results show that when compared with the control group, in the model group, *Enterorhabdus* (*p* < 0.05), *Candidatus*_*Saccharimonas* (*p* < 0.01), and *Ruminococcaceae*_UCG-013/UCG-014 (*p* < 0.05) significantly decreased. However, *Escherichia-Shigella* and *Helicobacter* significantly increased (*p* < 0.01). Compared with the model group, the decoction group had significantly decreased *Romboutsia* (*p* < 0.05) and significantly increased *Subdoligranulum* (*p* < 0.05), *Enterorhabdus* (*p* < 0.05), and *Oligella* (*p* < 0.05). In the volatile oil group, *Candidatus*_*Saccharimonas* (*p* < 0.05) and *Ruminococcaceae*_UCG-014 (*p* < 0.01) significantly increased and *Aerococcus* (*p* < 0.05), *Butyricicoccus* (*p* < 0.05), and *Romboutsia* (*p* < 0.01) significantly decreased. In the positive group, *Aerococcus* (*p* < 0.01), *Ruminococcaceae*_UCG-013 (*p* < 0.01), and *Jeotgalicoccus* (*p* < 0.01) significantly decreased, whereas *Anaerostipes* (*p* < 0.01) and *Enterorhabdus* (*p* < 0.05) significantly increased.

### 3.7. Analysis of Significant Difference between Samples

**Figure 8:**
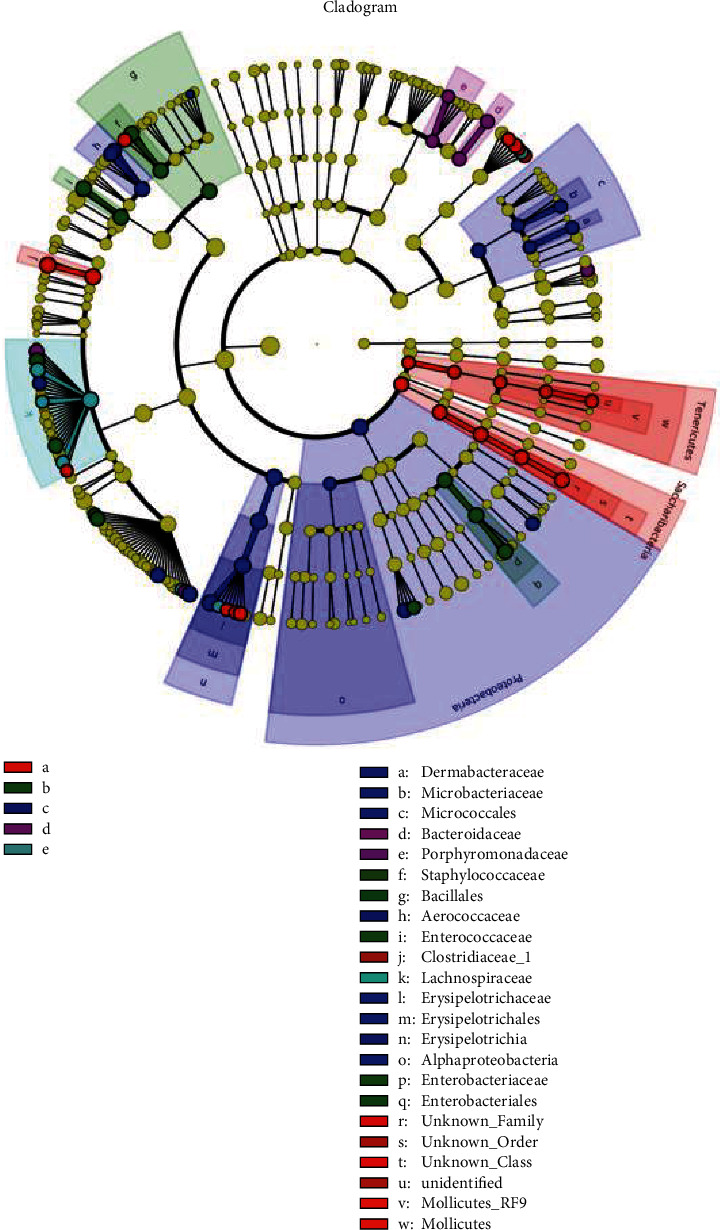
Cladogram: analysis of significant difference between samples: a—CG group, b—MG group, c—TG group, d—HG group, and e—YXG group.

The circle radiating from the inside to the outside represents the classification level from the door to the genus (or species). Each small circle at different levels represents a classification at this level, and the diameter size of the small circle is proportional to the relative abundance size of each genus or species (Figure 8). With coloring principle, the species without significant difference is colored yellow; the different species biomarkers follow the group coloring: the red node represents the microbial group that plays an important role in the “red group” and the green node represents the microbial group that plays an important role in the “green group.” The name of the species is represented in the English alphabet in the figure and is shown in the legend on the right.

**Figure 9:**
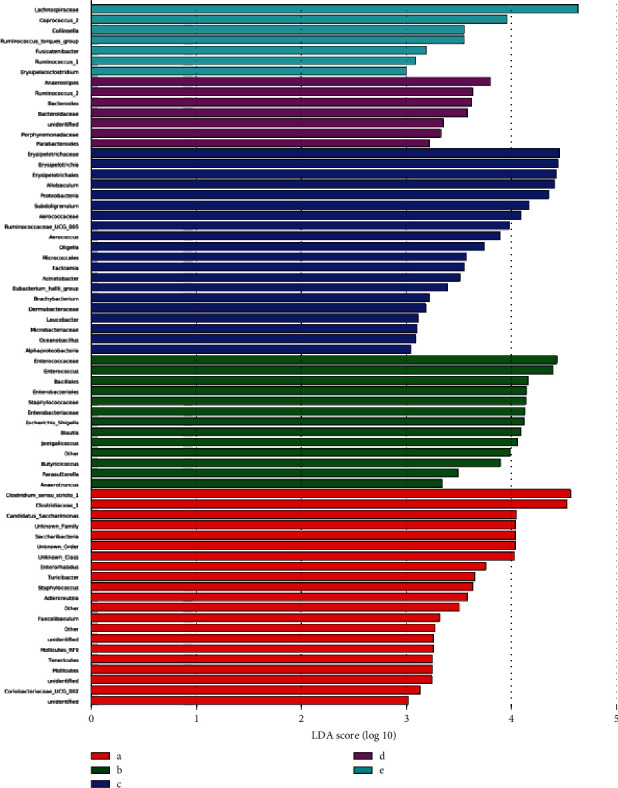
LDA score of significantly different bacteria in each group: a—CG group, b—MG group, c—TG group, d—HG group, and e—YXG group.

Figure 9 shows the species in which the linear discriminant analysis (LDA) score is greater than the set value, that is, the biomarker with statistically significant differences. This figure indicates species with significant differences in abundance in different groups, and the length of the histogram represents the size of the significantly different species. The longer the column, the greater the LDA value. *Clostridium*, *Clostridiaceae*, *Enterorhabdus*, *Staphylococcus*, soft-wall fungus door *Tenericutes*, and *Coriobacteriaceae* had the highest abundance in the control group. *Enterococcaceae*, *Bacillales*, *Enterobacteriales*, *Escherichia*, *Blautia*, and the genus *Anaerotruncus* were the most abundant in the model group. *Erysipelotrichia*, *Erysipelotrichaeae, Erysipelotrichichales*, *Allobaculum*, *Proteobacteria*, *Aerococcaceae*, *Ruminococcaceae*, *Aerococcus*, *Oligella*, *Micrococcales*, *Eubacterium Hallii*, *Brachybacterium*, *Dermabacteraceae*, *Microbacteriaceae*, marine *Oceanobacillus*, and *α-Proteobacteria* had the highest abundance in the decoction group. *Anaerostipes*, *Ruminococcus 2*, *Bacteroides*, *Bacteroidaceae*, *Porphyromonadaceae*, and *Parabacteroides* had the highest abundance in volatile oil groups. The different species with the highest abundance in the positive group were *Lachnospiraceae*, *Coprococcus*, and *Ruminococcaceae*.

## 4. Discussion

To test our hypothesis that regulation of intestinal microbiota by the Caoguo-4 decoction volatile oil is related to its antidiarrheal efficacy, we established the SDD model in rats demonstrated by indices of diarrhea and clinical symptoms, which is consistent with previous studies. Its main function is to affect the power of the gastrointestinal tract and can stimulate the secretion of pepsin and pancreatic fluid along with gallbladder contraction. The effect on gastrointestinal movement is manifested in increasing the tension of the lower esophageal sphincter, promoting the movement of the stomach, small intestine, and gallbladder, and contributing to the absorption of food. The experimental results showed that the plasma motilin in the model group was significantly higher than that in the control group, which was consistent with the previous studies such as Lu [16] and Li and Zhen [17], but the results are inconsistent with studies from groups such as Song et al. [18] and Shao et al. [19]; hence, there is a need to further study its mechanism. After administration with either the decoction group or volatile oil group, there is significant decrease in motilin, and the decrease in the volatile oil group was more obvious thus proving that this group was most efficient for the treatment of SDD. Results also showed that there was some damage to the intestinal mucosal structure in the model group, and the Caoguo-4 decoction and the Caoguo-4 decoction volatile oil group could improve the damaged epithelial structure and reduce inflammation.

The results of OTU analysis and alpha diversity analysis showed that the intestinal microbiota of spleen deficiency model rats changed significantly. With the increase in the number of samples and their corresponding grade values, the number of bacteria and relative abundance tend to be more towards a certain constant value. This indicates that the sequencing depth is sufficient to reflect the level of community richness, the number of OTUs is close to the actual situation, and the uniformity of community composition is higher. Additionally, the coverage value is the coverage of the sample library in the experiment, with 10% as the upper limit; the higher the value, the higher the probability of the sequence being detected in the sample, which means that the sequencing results can reflect the real situation of the sample.

Further, the richness and diversity of intestinal microbiota also decreased significantly. Through intervention with decoction and volatile oil, the richness and diversity of intestinal flora recovered significantly, but there was no significant change in the positive group. This shows that the intestinal microbiota in rats with SDD has been destroyed, and both decoction and volatile oil can restore the unbalanced intestinal microbiota, and it can be speculated that decoction and volatile oil are superior to Smecta in the treatment of spleen deficiency diarrhea.

Intestinal microbiota are the most important and diverse microbial community living in the human body [20], and in order to maintain the body function in a stable state, intestinal microbiota will restrict each other to achieve dynamic balance. Intestinal microbiota disorders in the TCM are considered important biological basis for “spleen deficiency” [21]. Further, the intestinal microecological changes of the SDD model are closely related to spleen inactivation. In this study, the thick-wall fungus door accounted for a large proportion in the gut, but there was no significant difference between the groups in this experiment. The change of the main intestinal microbiota caused by SDD is the decrease in the number of bacteria and *Saccharibacteria* in the intestinal tract (Bacteroidetes), but the number of bacteria in the deformed fungus door (Proteobacteria) increased correspondingly. This change was mainly due to the high nutritional requirements of the anaerobic bacteria Gram-negative *Bacillus* (mostly *Bacillus*-gate bacteria) caused by intestinal microenvironment destruction and the proliferation of lactobacillus bacteria with strong antibiotic resistance and lower nutritional requirements [22]. In this experiment, the *Proteobacteria* in the diarrhea model group of SDD was significantly increased, and the treatment of volatile oil in Caoguo-4 decoction inhibited the *Proteobacteria* in rats, and the bacteria of *Bacillus* were recovered. In this paper, the number of *Mycobacterium* doors in the model group was lower than that in the control group, and the number of *Bacillus* in the volatile oil group was the best to recover.

At the level of diarrhea, the genus *Escherichia coli*, *Enterococcus*, and *pylori* were observed to be increased, but the genus *Bifidobacterium* and rumen were reduced. *Escherichia coli* is the main pathogen causing the global epidemic of infectious diarrhea and outbreak of local diarrhea. The genus *Enterococcus* is a gram-positive coccus, which is aerobic or anaerobic and is in control of the host. Under controlled circumstances, it will not endanger the health of the host, but in the lack of nutrients, high alkaline and other harsh environment for a prolonged time can cause pathological changes, leading to infection disease [23, 24]. Bifidobacterium is a probiotic in the gut of humans and animals. It participates in a series of physiological processes, such as immunity, nutrition, digestion, and protection, and has the function of maintaining the balance of controlling intestinal microbiota, inhibiting the growth of pathogenic bacteria, and preventing and controlling constipation, dysentery, and gastrointestinal disorders [20]. In this study, each drug group had a conditioning effect on lactic acid bacilli in the gut of rats in the model group.

In addition, the regulation degree of the Caoguo-4 decoction agent and volatile oil on intestinal microbiota of SDD model rats was different. Compared with decoction, volatile oil had a conditioning effect on rumen fungus, pseudo-rod bacteria, and *Saccharibacteria*. Compared with the volatile oil group, decoction has a conditioning effect on *Bacillus*, *Enterococcus*, *Romboutsia*, *Subdoligranulum*, *Enterorhabdus*, and *Oligella*. The results show that the potential mechanism of the Caoguo-4 decoction agent and volatile oil on the SDD rat model is different, which may suggest the unique regulation mechanism of volatile oil on intestinal microbiota. In short, the two intervention groups changed the structure of intestinal microbiota and increased the beneficial bacteria such as the *Bifidobacterium* genus, rumen fungus, *Arabidopsis* bacteria, and *Saccharibacteria* and reduced harmful bacteria such as deformed fungus doors, *Escherichia coli*, *Romboutsia*, and *Blautia*. Further, by analyzing the species or communities with significant differences between the groups, it was found that the changes of *Enterococcus*, *Enterobacter*, *and Escherichia coli* may be related to the diarrhea index of spleen deficiency.

## 5. Conclusion

In summary, Caoguo-4 decoction and volatile oils are beneficial against diarrhea, which reduced the incidence of diarrhea and improved stool. Our research proved that Caoguo-4 decoction and volatile oils can upregulate the content of D-xylose, AML, and MTL in rats, changing the structure of intestinal microbiota by increasing the beneficial bacteria such as the *Bifidobacterium* genus, rumen fungus, *Arabidopsis bacteria*, and *Saccharibacteria* and reducing harmful bacteria such as deformed fungus doors, *Escherichia coli*, *Romboutsia*, and *Blautia*. To achieve treatment of spleen deficiency diarrhea with its mechanism being still unknown, further research is needed.

## Figures and Tables

**Table 1:** The contents of D-xylose, AML, and MTL in blood of rats with spleen deficiency diarrhea.

Group	D-Xylose	AMS	MTL
CG	1.66 ± 0.30	4294 ± 848	167.58 ± 25.73
MG	0.81 ± 0.37^∗^	2469 ± 929^∗^	196.34 ± 23.47^∗^
TG	2.52 ± 1.20^#^	2880 ± 371	160.47 ± 28.68^#^
HG	2.15 ± 0.41^##^	3862 ± 559^#^	145.20 ± 17.99^##^
YXG	2.07 ± 0.54^##^	3712 ± 740^#^	202.03 ± 62.39

Note: CG: control group; MG: model group; TG: soup group; HG: oil group; YXG: positive group. Compared with the control group, ^∗^*p* < 0.05, ^∗∗^*p* < 0.01; compared with the model group, ^#^*p* < 0.05, ^##^*p* < 0.01.

**Table 2:** Comparison of OTUs and diversity of samples between groups.

Groups	*A*	Observed species	PD-whole tree	*B*	Recovery rate
CG	449.86 ± 28.72	351.50 ± 10.64	34.48 ± 0.31	4.47 ± 0.43	>0.99
MG	286.88 ± 22.05^∗∗^	233.80 ± 13.62^∗∗^	24.40 ± 1.89^∗∗^	4.59 ± 0.15	>0.99
TG	393.16 ± 36.37^##^	311.08 ± 34.21^##^	30.42 ± 3.02^#^	5.03 ± 0.47	>0.99
HG	379.11 ± 58.53^#^	312.05 ± 55.76^#^	30.79 ± 4.18^#^	4.47 ± 0.89	>0.99
YXG	337.04 ± 61.82	274.28 ± 39.38	28.17 ± 3.04	4.57 ± 0.42	>0.99

Note: CG: control group; MG: model group; TG: soup group; HG: oil group; YXG: positive group. Compared with the control group, ^∗^*p* < 0.05, ^∗∗^*p* < 0.01; compared with the model group, ^#^*p* < 0.05, ^##^*p* < 0.01.

**Table 3:** Species at different groups of phylum classification levels (%).

Phylum	CG group	MG group	TG group	HG group	YXY group
*Cyanobacteria*	0.12%	0.00%^∗∗^	0.03%	0.01%	0.01%
*Saccharibacteria*	2.30%	0.00%^∗^	0.10%^#^	0.07%^#^	0.14%
*Proteobacteria*	1.36%	5.24%^∗∗^	5.89%	3.97%^#^	3.96%
*Tenericutes*	0.42%	0.05%^∗∗^	0.10%	0.15%	0.09%
*Bacteroidetes*	2.17%	0.64%	1.57%	5.47%	1.24%
*Firmicutes*	78.76%	84.05%	80.95%	81.78%	86.42%
*Euryarchaeota*	0.04%	0.00%^∗^	0.01%	0.01%	0.01%
*Verrucomicrobia*	0.21%	0.37%	0.36%	0.64%	1.09%
*Actinobacteria*	14.54%	9.61%	10.96%	7.90%	7.03%
Other	0.07%	0.03%	0.02%	0.02%	0.01%

Note: compared with the control group, ^∗^*p* < 0.05, ^∗∗^*p* < 0.01; compared with the model group, ^#^*p* < 0.05, ^##^*p* < 0.01.

**Table 4:** Species at different groups of genus classification levels (%).

Genus	GC	MC	TJG	HFYG	YXZ
*Collinsella*	0.01%	0.55%^∗∗^	0.07%^##^	0.64%	0.74%
*Phascolarctobacterium*	0.00%	0.65%^∗^	0.08%^##^	0.19%	0.17%
*Bifidobacterium*	3.10%	1.14%	1.20%	1.80%	1.16%
*Akkermansia*	0.21%	0.37%	0.36%	0.64%	1.09%
*Ruminococcaceae_UCG-014*	3.32%	0.95%^∗^	2.49%	3.35%^##^	2.09%
*Anaerostipes*	0.00%	0.33%	0.09%	1.27%	1.21%^##^
*Acinetobacter*	0.00%	0.11%^∗^	0.73%	0.03%	0.30%
*Ruminococcaceae_UCG-013*	0.87%	0.13%^∗^	1.15%	0.55%	0.78%^#^
*Ruminococcus_torques_group*	0.00%	0.42%^∗^	0.19%	0.11%	0.74%
*Escherichia-Shigella*	0.00%	2.43%^∗∗^	1.71%	2.16%	2.31%
*Aerococcus*	1.36%	0.85%	1.55%	0.21%^#^	0.23%^#^
*Anaerotruncus*	0.01%	0.54%	0.19%	0.37%	0.15%
*Bacteroides*	0.00%	0.20%^∗∗^	0.07%^#^	0.78%	0.25%
*Faecalibaculum*	0.42%	0.02%^∗∗^	0.17%	0.13%	0.05%
*Butyricicoccus*	0.01%	1.80%^∗^	0.79%	0.12%^#^	0.53%
*Staphylococcus*	0.83%	0.44%	0.19%	0.02%^#^	0.10%
*Desulfovibrio*	0.49%	0.22%	0.34%	0.11%	0.06%
*Jeotgalicoccus*	1.10%	2.79%	1.72%	0.53%^#^	0.77%^#^
*Eubacterium_coprostanoligenes_group*	0.23%	0.11%	0.52%	0.67%	0.65%
*Romboutsia*	10.56%	8.65%	6.03%^#^	3.83%^##^	6.92%
*Blautia*	0.03%	2.52%^∗^	2.23%^##^	1.15%	2.48%
*Subdoligranulum*	0.00%	0.62%^∗^	2.91%^#^	0.27%	0.66%
*Lachnospiraceae_NK4A136_group*	0.27%	0.09%	1.00%	0.05%	0.06%
*Lactobacillus*	40.83%	44.49%	37.52%	53.23%	52.16%
*Candidatus_Saccharimonas*	2.28%	0.00%^∗∗^	0.05%	0.07%^#^	0.12%
*Oligella*	0.52%	0.20%	1.44%^#^	0.10%	0.37%
*Allobaculum*	6.17%	7.75%	8.52%	4.09%	3.27%^#^
*Enterococcus*	0.23%	4.47%	4.30%	2.21%	2.42%
*Facklamia*	0.48%	0.43%	0.85%^#^	0.10%^#^	0.25%^#^
*Ruminococcus_2*	0.05%	0.11%	0.15%	0.91%^#^	0.79%^#^
*Adlercreutzia*	0.93%	0.22%	0.13%	0.12%	0.17%
*Enterorhabdus*	1.55%	0.27%^∗^	0.54%^#^	0.42%	0.48%^#^
*Helicobacter*	0.00%	1.25%^∗∗^	0.97%	1.09%	0.56%
*Eubacterium_hallii_group*	0.00%	0.34%^∗^	0.51%	0.08%	0.31%
*Parasutterella*	0.00%	0.74%	0.15%	0.42%	0.13%
*Corynebacterium_1*	6.67%	5.78%	6.60%	2.95%	3.19%
*Ruminococcaceae_UCG-005*	0.08%	0.17%	2.22%	1.64%	1.74%
*Clostridium_sensu_stricto_1*	7.27%	0.07%	0.01%	1.97%^#^	0.67%^#^
Other	10.08%	7.77%	9.66%	11.04%	9.24%

Note: CG: control group; MG: model group; TG: soup group; HG: oil group; YXG: positive group. Compared with the control group, ^∗^*p* < 0.05, ^∗∗^*p* < 0.01; compared with the model group, ^#^*p* < 0.05, ^##^*p* < 0.01.

## Data Availability

All data used to support the findings of this study are available from the corresponding author upon request.
